# Socioeconomic, Psychiatric and Materiality Determinants and Risk of Postpartum Depression in Border City of Ilam, Western Iran

**DOI:** 10.1155/2013/653471

**Published:** 2013-07-24

**Authors:** Pegah Taherifard, Ali Delpisheh, Ramin Shirali, Abdorrahim Afkhamzadeh, Yousef Veisani

**Affiliations:** ^1^Department of Anesthesiology, Gilan University of Medical Sciences, P.O. Box 41938-93345, Rasht, Iran; ^2^Department of Clinical Epidemiology, Ilam University of Medical Sciences, P.O. Box 69315-138, Ilam, Iran; ^3^Prevention of Psychosocial Injuries, Research Centre, P.O. Box 69315-138, Ilam, Iran; ^4^Department of Community Medicine, Kurdistan University of Medical Sciences, P.O. Box 66177-13446, Sanandaj, Iran; ^5^Student Research Committee, Ilam University of Medical Sciences, P.O. Box 69311-57793, Ilam, Iran

## Abstract

*Background*. Postpartum depression (PPD) is considered as one of the mood disturbances occurring during 2-3 months after delivery. The present study aimed to determine the prevalence of PPD and its associated risk factors in border city of Ilam, western Iran. *Methods*. Through a descriptive cross-sectional study in 2011, overall, 197 women who attended Obstetrics & Gynecology clinics postpartumly in the border city of Ilam, western Iran, were randomly recruited. A standard questionnaire that was completed by a trained midwife through face to face interviews was used for data gathering. *Results*. Mean age ± standard deviations was 27.9 ± 5.2 years. Prevalence of PPD was estimated to be 34.8% (95% CI: 27.7–41.7). A significant difference was observed among depression scores before and after delivery (*P* ≤ 0.001). Type of delivery (*P* = 0.044), low socioeconomic status (*P* = 0.011), and women having low educational level (*P* = 0.009) were the most important significant risk factors associated with PPD. The regression analysis showed that employed mothers compared to housekeepers were more at risk for PPD (adjusted OR = 2.01, 95% CI: 1.22–2.28, *P* = 0.003). *Conclusions*. Prevalence of PPD in western Iran was slightly higher than the corresponding rate from either national or international reports.

## 1. Introduction 

Pregnancy, delivery, and adaptability with newborn babies are the most sensitive phases in women's life [1]. In this period of time, women usually suffer from physiological, spiritual, and psychological crises leading to change of their positions. The first six weeks after delivery is a vulnerable period for postpartum depression (PPD) [2]; of 4 million births that occur in the world annually, approximately 40 percent of new mothers are affected with different types of postpartum mood disorders including depression symptoms before and during pregnancy [3]. Prevalence of PPD is closely linked with socioeconomic and cultural factors and it varies among different countries, ethnicities, and races [4]. The global prevalence of PPD has been estimated to be between 10–25 percent [5–7] and between 27–39 percent in Iran [8, 9]. 

Various putative psychosocial and obstetric factors have been studied and suggested as risk factors for the development of PPD; if these results are inconsistent and do not effectively help predict women at risk, knowledge of these factors may help identify those who are at higher risk and can benefit from early professional help [10]. Personal history of depression (prior to pregnancy or postpartum) is the major risk factor for PPD [11–14]. Family psychiatric history [15], lack of perceived social support for the pregnancy from family and friends [16, 17], unemployment of the mother or head of the household [18], lack of emotional and financial support from the partner [19], marital conflict [20], stressful life events in the previous 12 months [21], living without a partner [21], unplanned pregnancy [22], not breastfeeding [23], childcare-related stressors [23], sick leave during pregnancy related to uterine irritability, psychiatric disorders, high number of visits to prenatal clinic [24], and a congenitally malformed infant [25], are other risk factors of PPD.

Due to adverse effects of increased PPD prevalence in Iran, all health care workers and nurses in particular should be able to manage postpartum psychological disorders. The present study aimed to determine prevalence of PPD and its associated risk factors in the border city of Ilam, western Iran.

## 2. Methods

Through a descriptive cross-sectional study in 2009, overall, 215 women who attended ten obstetrics and gynecology clinics postpartumly in the border city of Ilam, western Iran, were randomly selected, of which 197 women (90.2%) were recruited. All women gave their informed consent. A standard questionnaire containing Socioeconomic factors and psychiatric and materiality characteristics was completed by a trained midwife. Face to face interviews were used for data gathering. Interviews took place between the 6th and the 8th week after delivery from 10 March 2009 to 20 July 2009. Due to methodological issues, such as questionnaire errors or missing data, 18 (9.8%) women were excluded.

The main outcome of the present study was PPD assessed by the use of the Edinburgh postpartum depression scale [26, 27]. The scale consisted of 10 questions with four response categories scored from 0 to 3, whereby the greatest values represent depressed moods. Mothers who obtained an Edinburgh postpartum depression scale total score of 13 or greater were labeled as having PPD [28]. Score of 0–9 inclusively indicates no risk of experiencing symptoms of PPD, a score of 10–12 indicates a minor/major risk of experiencing symptoms of PPD; and a score of 13 or greater indicates a major risk of experiencing symptoms of PPD [29]. The sensitivity and specificity of the EPDS have been found to be 75% and 97% respectively, reported in Persian version at a cutoff of 13 [30] and have been used and found to be valid in Iranian studies [30–32]. Major depression is defined as a clinical syndrome that has a clinical treatment process. 

 Socioeconomic factors, such as maternal educational level (illiterate, less than high school (primary), diploma, university graduate), household income by Iranian toman per month (low < 400.000, middle 401.000–600.000, high > 601.000), occupation during pregnancy (housekeeper, employed), and partners' occupation (unemployed, employed), were examined. Information about maternal characteristics including parity, type of delivery (Cesarean section, normal vaginal), pregnancy weight gain guidelines (inadequate, recommended, excessive), family planning (all pregnancies, unplanned), and psychiatric determinants like previous diagnosis of depression/prescription antidepressants, mother's stress level during pregnancy (very, somewhat, no), satisfaction from living with husband (very high, moderate, and very poor), and satisfaction from living with husband/partner (very high, moderate, very poor) were explored. All the variables were directly self-reported by the mother. Household income was calculated based on the number of people in the household and the total household income before taxes and deductions earned by all household members from all sources in the past one year. A mother's stress level during pregnancy was based on the amount of stress reported during the one year prior to the baby's birth. A logistic regression model was used to compute the odds ratios (ORs) for dependent risk factors associated with PPD. To demonstrate the initial results, univariate ORs with 95% confidence intervals (CIs) for demographic variables and psychological risk factors were conducted. A multiple logistic regression analysis was executed to detect PPD as the dependent variable and risk factors as independent variables. SPSS version 16 was used for all analyses. Probability values equal or less than 0.05 were considered statistically significant.

## 3. Results

 Mean maternal and paternal ages were 27.9 ± 5.2 years and 35.4 ± 27.6 years, respectively. The mean maternal marriage age was 22.3 ± 4.1 years. Majority of husbands had informal jobs and small business (60.4%). Mean duration of hospitalization for delivery was 1.96 ± 0.28 days, and the mean birth weight was 3344 ± 384 grams. More than half of mothers were satisfied (54.8%), and their pregnancies were previously planned. One in every five pregnancies (20.3%) was totally unplanned. Prevalence of severe maternal sadness during pregnancy was 6.1%, and 5.6% of mothers had a severe form of grief and depression after pregnancy. Almost one fourth of mothers (24.4%) had consumed medication(s) during pregnancy. Unpleasant and stressful life events occurred in 10.7% of cases. Severe mental disorders had occurred for 6.6% of cases. Prevalence of PPD was 34.8% (95% CI: 27.7–41.7). However, almost all mothers (99.5%) had no history of PPD, but 2.5% of partners had a history of mental disorders. Method of delivery in 52.3% of cases was Cesarean section. In general, 91.9% of mothers had a term delivery and 97% had no delivery complications. Almost all (95.4%) of the newborns were breastfed. 

In Table 1, we present results of the logistic regression model for each variable and univariate ORs with 95% CIs risk of depression in postnatal period by socioeconomic characteristics. Compared with employment, unemployment was significantly related to a reduced risk of PPD and women whose husbands were employed (OR = 1.03, 95% CI: 0.87–1.44); both differences were statistically no significant. We also found a significant declining trend by economic status. Women with low economic status had a more than two times higher risk of depression than those with high economic status (OR = 2.45, 95% CI: 1.56–4.13).

We observed significant results for the psychiatric determinants risk factors (Table 2). Women with poor family relationships in their current family had a higher risk of depression (OR = 1.37, 95% CI: 1.07–1.92). Significant excess risk also appeared with insufficient family support during the pregnancy (OR = 2.03, 95% CI: 1.16–3.31), and we also observed an excess risk of depression among women with emotional stress during the pregnancy (OR = 2.61, 95% CI: 1.67–3.11). 

Adjusted ORs by materiality factors are presented in Table 3. There was a significant excess risk of depression for women who have Caesarean section (OR = 1.66, 95% CI: 1.09–2.0). There was a significant excess risk of depression for unplanned pregnancies (OR = 2.11, 95% CI: 1.44–2.56). Women who had a parity 3 ≤ (OR = 1.41, 95% CI: 1.18–2.03), showed an excess risk of depression.

 Based on the likelihood ratio test, risk factors such as education, occupation, history of depression, parity, and type of delivery were kept in the final multilogistic regression model (Table 4). The regression analysis showed that employed mothers compared to housekeepers were more at risk for PPD (adjusted OR = 2.01, 95% CI: 1.22–2.28, *P* = 0.003). After adjusting for the other independent variables, a prior diagnosis of mild and moderate/severe depression remained significant for major PPDs (OR: 2.61, 95% CI: 1.67–3.11 and OR: 2.10, 95% CI: 1.37–2.81, resp.). The ORs and 95% CIs of the ORs and probability of the significant level for the risk variables are shown in Table 2. All reference categories had ORs = 1.

## 4. Discussion 

**Figure 1:**
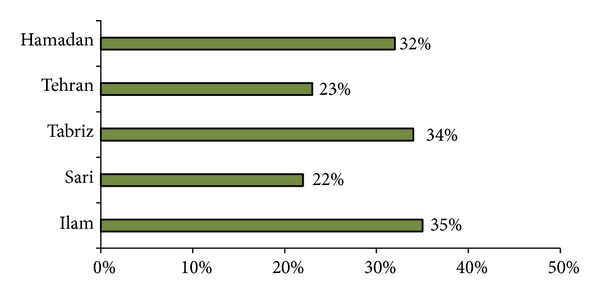
Prevalence of PPD in different cities in Iran.

**Figure 2:**
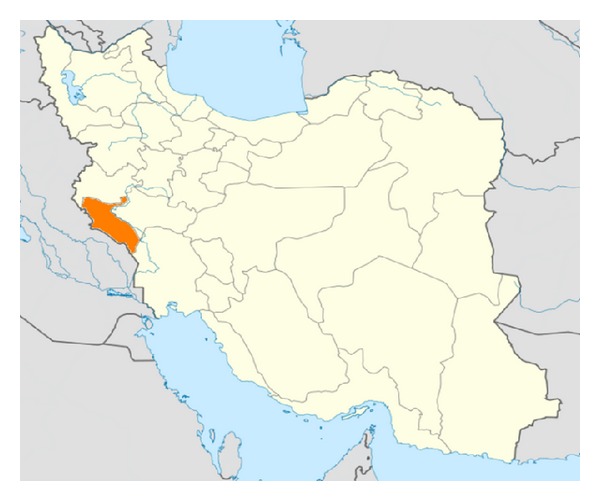
Map of study setting, Ilam, Iran.

In the present study, the prevalence of PPD was 34.8%. In the present study, the prevalence of PPD was 34.8%. In a similar study conducted in Shiraz city southern Iran 20.3%, [20] corresponding rate was 22% in Sari city northern, [33] 34% in Tabriz city northwest, [34] 23% in Tehran capital, [35] and 32% in Hamadan city western the country [36]. (Figure 1). This result is similar to other reports in Middle East developing countries such as Iraq, Erbil city, Kurdistan region prevalence of PPD was 28.4% [37], and 37.1% in Bahraini women [38]. In African countries (Nigerian women 27. 2%) [39] and South America (Brazilian women 24.3%) [40], similar result has been reported. The present study was undertaken in Ilam city, western Iran, and nearby Iraqi border. Participants were mothers who almost gave birth during the eight-year bloody war of Iraq against Iran starting from 1981 onward. So they have seen all war-related problems and difficulties directly and therefore this may have effects on high prevalence of PPD in this region (Figure 2). Of course we are not claiming that increased PPD in border cities is solely due to the war issue, but this could be one of several known and unknown risk factors. However, differences in PPD among regions or countries can be partially explained by the effect of income on the mediation of risk factors.

Previous studies in different parts of Iran have shown significant association between PPD and age of partner, [33] socioeconomic status [20], number of children [8], parity [8], and pregnancy interval [32]. The current study showed that, compared with unemployment, employment was significantly associated with a positive risk of PPD. There were relationships between household income or educational levels and the risk of postpartum depression. A study in India showed a significant positive relationship between employment and PPD [41]. Null findings regarding employment have been observed in studies conducted in the United States [17], Brazil [42], Turkey [43], and Sweden [23]. The current findings are in agreement with these observations. In contrast, significant positive association was observed between unemployment and postpartum depression in research conducted in France [44], Sweden [24], Turkey [45], Ireland [46], and the United Kingdom [22]. The significant positive association that we observed between employment and PPD was likely to be ascribed to job stress and its limitations for women in Iran. In a meta-analysis of 46 studies, employees with low levels of job satisfaction were more likely to have raised levels of depression [18]. 

 No relationship was found between household income and PPD in the United States [17] and Australia [47]. The present results are variant with these findings but consistent with those reports of a positive association between low income and PPD in research that was conducted in the United States [5, 16, 19], Brazil [42], and Turkey [48]. Our finding of a positive association between educational level and PPD disagrees with results of research in the United States [13, 17], Brazil [42], Turkey [43], Australia [47], and Italy [6]. However, studies conducted in the United States [19], Turkey [45], and India [41] reported that a low educational level was positively related to the risk of PPD.

The present study was limited by using a questionnaire to determine PPD. The diagnosis of PPD was established with the EPDS, a self-report rating scale, rather than a clinician-administered structured diagnostic interview. Moreover, the validity and reliability of such tools have already been documented [30]. Due to significant association between prenatal and postnatal maternal depressions, it can be recommended that the Edinburgh questionnaire should routinely be used in the third trimester of pregnancy to diagnose susceptible mothers and to undertake preventive strategies and controlling approaches.

In conclusion, prevalence of PPD in western Iran was slightly higher than the corresponding rate from either national or international reports which could be in part due to socioeconomic status or cultural differences. Women should be routinely evaluated for postnatal depression, and those with lower education or income are likely to require further care from health services and should be given the benefit of mental health prevention programs. However, promotion of education and increasing the social welfare result in improving the socioeconomic program and may reduce the risk of PPD. 

## Figures and Tables

**Table 1:** Univariate odd ratios (ORs) for sociodemographic characteristic risk factors of postpartum depression.

Factors	Responses (%)	OR (95% CI)	*P* value
Socioeconomic status (Iranian toman/month)		Overall	0.011
Low (<400.000)	45.7	2.45 (1.56–4.13)	
Moderate (401.000–600.000)	45.3	1.77 (1.22–2.39)	
High (>601.000)	9.0	1	
Mothers' educational level		Overall	0.009
Illiterate	1.5	1.29 (1.11–1.77)	
Primary (less than high school)	56.5	1.17 (1.07–1.45)	
Diploma	42.0	1.03 (0.77–1.42)	
University graduate	2	1	
Mothers' occupation		Overall	0.008
Housekeeper	86.3	1	
Employed	13.7	2.01 (1.22–2.88)	
Partners' occupation		Overall	0.212
Unemployed	39.6	1	
Employed	60.4	1.03 (0.87–1.44)	
Partners' age		Overall	0.444
<25	32.1	1	
25–35	35.9	1.15 (1.00–1.67)	
>35	32.0	1.23 (1.03–1.73)	

**Table 2:** Univariate odd ratios (ORs) for psychiatric risk factors of postpartum depression.

Factors	Responses (%)	OR (95% CI)	*P* value
History of infertility		Overall	0.113
All pregnancies	24.9	1	
Planned	54.8	0.66 (0.19–1.02)	
Unplanned	20.3	2.11 (1.44–2.56)	
Receiving family support during pregnancy		Overall	0.042
Yes/always	46.7	1	
No/occasionally	53.3	2.03 (1.16–3.31)	
Mother's stress level during pregnancy		Overall	0.085
Very	21.1	1.01 (0.88–1.13)	
Somewhat	40.9	1.06 (0.67–1.27)	
No	39.0	1	
History of depression during pregnancy		Overall	0.002
Mild	42.1	2.61 (1.67–3.11)	
Moderate/severe	5.6	2.10 (1.37–2.81)	
No/never	52.3	1	
Satisfaction from living with husband		Overall	0.214
Very high	46.7	0.66 (0.25–0.87)	
Moderate	11.2	1.28 (1.00–1.55)	
Very poor	42.1	1	

**Table 3:** Univariate odd ratios (ORs) for materiality risk factors of postpartum depression.

Factors	Responses (%)	OR (95% CI)	*P* value
Family planning		Overall	0.113
Planned	54.8	1	
Unplanned	55.2	2.11 (1.44–2.56)	
Parity		Overall	0.084
1	6.1	1	
2	32.5	1.35 (1.01–1.89)	
≥3	61.4	1.41 (1.18–2.03)	
Type of delivery		Overall	0.044
Cesarean section	52.3	1.66 (1.09–2.00)	
Normal vaginal	47.7	1	
Pregnancy weight gain guidelines		Overall	0.240
Inadequate	32.1	1	
Recommended	60.5	1.01 (0.89–1.11)	
Excessive	7.4	1.03 (0.66–1.31)	

**Table 4:** Adjusted odds ratios (ORs) from the multiple logistic regression analysis of postpartum depression risk factor.

Factors	Responses (%)	OR (95% CI)	*P* value
Educational levels		Overall	<0.001
Illiterate	1.5	1.29 (1.11–1.77)	0.002
Primary	56.5	1.17 (1.07–1.45)	0.125
Diploma	42.0	1.03 (0.77–1.42)	0.002
University graduate	2	1	
Job status		Overall	<0.001
Housekeeper	86.3	1	
Employed	13.7	2.01 (1.22–2.88)	0.003
Receiving family support during pregnancy		Overall	<0.001
Mild	42.1	2.61 (1.67–3.11)	0.009
Moderate/severe	5.6	2.10 (1.37–2.81)	<0.001
No/never	52.3	1	
Parity		Overall	0.004
1	6.1	1	
2	32.5	2.35 (1.91–3.77)	0.224
≥3	61.4	3.41 (2.88–4.09)	0.008
Type of delivery		Overall	0.044
Cesarean section	52.3	1.66 (1.09–2.00)	0.003
Normal vaginal	47.7	1	
